# Big Data: the challenge for small research groups in the era of cancer genomics

**DOI:** 10.1038/bjc.2015.341

**Published:** 2015-10-22

**Authors:** Aisyah Mohd Noor, Lars Holmberg, Cheryl Gillett, Anita Grigoriadis

**Affiliations:** 1 Research Oncology, Faculty of Life Sciences and Medicine, King's College London, Guy's Hospital, London SE1 9RT, UK; 2 Department of Surgical Sciences, Uppsala University, Uppsala 751 85, Sweden; 3 Faculty of Life Sciences and Medicine, King's Health Partners Cancer Biobank, King's College London, Research Oncology, Guy's Hospital, London SE1 9RT, UK; 4 Breast Cancer Now Research Unit, Research Oncology, Faculty of Life Sciences and Medicine, King's College London, Guy's Hospital, London SE1 9RT, UK

**Keywords:** cancer research, database management systems, biobanking, genomics, ontology management, data ethics

## Abstract

In the past decade, cancer research has seen an increasing trend towards high-throughput techniques and translational approaches. The increasing availability of assays that utilise smaller quantities of source material and produce higher volumes of data output have resulted in the necessity for data storage solutions beyond those previously used. Multifactorial data, both large in sample size and heterogeneous in context, needs to be integrated in a standardised, cost-effective and secure manner. This requires technical solutions and administrative support not normally financially accounted for in small- to moderate-sized research groups. In this review, we highlight the Big Data challenges faced by translational research groups in the precision medicine era; an era in which the genomes of over 75 000 patients will be sequenced by the National Health Service over the next 3 years to advance healthcare. In particular, we have looked at three main themes of data management in relation to cancer research, namely (1) cancer ontology management, (2) IT infrastructures that have been developed to support data management and (3) the unique ethical challenges introduced by utilising Big Data in research.

In the past decade there has been an unprecedented volume of data generation in cancer research. The emphasis on translational approaches in research, brought on by the need for accelerated, cost-effective research solutions has spurred a host of initiatives towards integration of multi-disciplinary clinical and research data to better inform research questions, including the Center for Advancing Translational Science under the National Institutes of Health (NIH, 2011) and the Cancer Research UK's Stratified Medicine Programme (Cancer Research UK, 2013). Furthermore, the development of high-throughput methods for genome interrogation, such as microarrays and next-generation sequencing (NGS), have allowed more in-depth study of tumour biology at the genetic and genomic level, leading to better targeted and personalised healthcare solutions for cancer patients. Taking breast cancer as an example, the ‘Big Data' revolution has given rise to a multitude of genome-driven molecular signatures with the potential to further personalise diagnosis and treatment (Dawson *et al*, 2013). An increasing number of signatures are being validated and adopted into standard practice such as the Oncotype DX (Paik *et al*, 2006) and Mammaprint (van't Veer *et al*, 2002) gene expression scores.

Although these developments have been crucial to improved cancer healthcare, they have presented a quandary to those at the heart of the growth of Big Data. Biological and clinical researchers now face increasingly large and complex data sets. Although a standard genomic microarray may profile a genome for hundreds to thousands of features per sample, current next-generation sequencers can produce over 100 GB of raw sequence reads per genome. These data, coupled with a plethora of clinical and phenotypic attributes, have the potential to significantly expand our understanding of disease. However, they also present non-trivial issues in data storage and analysis, issues which are relatively new in biomedicine compared with fields such as commerce and finance, where industrial-scale analyses of Big Data have been established for many years.

The relative lag in adopting Big Data in biomedicine can be attributed to three main factors: First, much of healthcare still relies on paper records and manual recording of data, despite increasing digitisation of health records, thereby leading to non-standardised, error-prone data recording (MacRury *et al*, 2014). Second, clinical and research data often exist in islands, separated by legal and intellectual property requirements, as well as security and confidentiality restraints (GA4GH, 2013). Third, the IT infrastructures available to researchers are ill-equipped to handle the integration and capture of heterogeneous and large-scale data, an issue that was acknowledged in a study of 17 leading academic health centres in the US (Murphy *et al*, 2012). At present, the real value lies not in reporting on data from these individual silos of information, but rather in the ability to bring these data together to find meaningful associations across multiple sources (Costa, 2014).

Overcoming these barriers requires the development of efficient database management systems (DBMS) that provide a centralised data source to consolidate disparate data sets. Such an example of large-scale, collaborative genomics study is the 100 000 Genomes Project, recently introduced by the Department of Health with the goal of sequencing the entire genomes of over 75 000 patients by 2017 to advance medical research and integrate genomics into healthcare (Genomics England, 2014). The project will leverage on collaboration between academic and commercial researchers registered in the Genomics England Clinical Interpretation Partnership (GeCIP) programme. However, such ventures raise ethical concerns regarding the flow of data and patient samples across the healthcare-research spectrum. Research groups involved in such studies will need to re-evaluate their current data management systems to adapt to these unique set of technical and ethical challenges.

## Data heterogeneity: one size does not fit all

Data standards in cancer research have evolved considerably in the past decade. Rapid developments in tumour classification and drug discovery are now overtaking the rate at which they are adopted into traditional vocabularies such as the International Classification of Diseases for Oncology (ICD; Mirnezami *et al*, 2012). The evolution of clinical data standards has been taxonomised by medical informaticians from as early as the 1990s, covering a broad range of semantic and syntactic transitions; examples of these are pre-coordination (from ‘Carcinoma *in situ* of the breast' in the ICD-9 to the child concepts of ‘Intraductal carcinoma *in situ* of unspecified breast', ‘Intraductal carcinoma *in situ* of right breast' and ‘Intraductal carcinoma *in situ* of left breast' in the ICD-10) and obsolescence of redundant concepts (Cimino, 1996). As databases rely on the accurate classification of data, these changes have substantial effects in the way databases are modelled and structured, and subsequently in how they are queried. Therefore, current databases have a strong need for cancer ontologies that can standardise data accurately.

The ISO/IEC 11179 standards for electronic data recording provide the basis for standard medical ontologies including the Systematised Nomenclature of Medicine Clinical Terms and the Health Level Seven International (HL7) protocol as well as cancer-specific ontologies such as the NIH Cancer Data Standards Registry and Repository (caDSR) and the National Cancer Institute (NCI) Thesaurus. To conform to these standards, a metadata model is typically constructed for the data set. This can be in the form of a hierarchical data structure such as the Unified Medical Language System (Humphreys *et al*, 1998) which is an NIH-based medical data structure that maps data to established ontologies like HL7 and ICD-10. Metadata can also be represented through an entity-attribute-value model, which classifies concepts into an element (e.g., patient), attribute (e.g., tumour grade) and a value domain defining the range of permissible values for the element (e.g., grade 1–3). The construction of metadata is an often laborious, costly and time-consuming step in database development and requires careful planning, evaluation of the research protocols involved in the study, consultation with end-users of the database, and in the case of multi-institutional studies, examination of the legacy data models already in place in each institution. For this reason, well-curated metadata models are characteristic of large, long term clinical studies including those developed for the Surveillance, Epidemiology and End Results Program (SEER, 2008) and the NCI's Clinical Trials Cooperative Group (Komatsoulis *et al*, 2008).

An alternative method which may be more suited to smaller or short-term studies, is the alignment of data sets to widely available common data element (CDE) models that have been readily mapped to the ontology of choice, for instance the LexEVS model referenced upon the Enterprise Vocabulary Services (Ethier *et al*, 2013), and the caGRID CDE referenced upon the caDSR metadata model (Papatheodorou *et al*, 2009). These models serve as a ‘mediator' by allowing different data sets to retain their legacy terms, while providing a unifying data model for the purposes of the study. Many other CDE mappings to ontologies have been developed and are summarised in the NIH BioPortal, which uses pattern-matching algorithms to generate mappings across >400 biomedical ontologies (Salvadores *et al*, 2013). The advantage to small research groups in this approach is the continuous, community-driven curation of these mappings, thus assuring ISO-compliant standardisation of their data sets at a cheap cost.

Besides manual curation of metadata, a common approach is the alignment of data sets to an ontology using natural language processing methods. Examples are the Dice and Dynamic algorithms that match element attributes to CDE element-attribute pairs (Ghazvinian *et al*, 2009) and the Apriori algorithm for automated identification of CDEs in medical records, as trained by human use (Luo *et al*, 2013). These methods, although dependent on established computing facilities, aid in automating the processing of free text into standardised classes of information, thereby reducing manual abstraction and human error.

Many of the proposed solutions have been generated through open-source initiatives and have been instrumental to the development of cancer databases and ontology-based applications, especially for smaller research groups. However, previous audits of commonly used cancer ontologies like the NCI Thesaurus have shown that they are far from perfect. A number of CDE's may be misclassified when matched to other ontologies (Jiang *et al*, 2012), and when a CDE model is rigidly enforced, may introduce inaccurate semantic mapping (Schulz *et al*, 2010). On one hand, this issue emphasises the requirement for periodic quality assurance of metadata models to maintain accuracy; on the other, it has prompted the practice of using less rigid data models where the database architecture is less sophisticated, as is the case in small-scale research. Community-driven efforts to develop and maintain the ontologies have been vital in keeping them up-to-date, and in sharing best practices for applying CDE normalisation to data sets (de Coronado *et al*, 2009). These approaches have shown that the over-arching goal is not to enforce an all-encompassing rule for the definition of data across studies, but rather to achieve a harmonisation of the consolidated data sets for the purposes of interoperability.

## Technical infrastructure—the base of the iceberg

NGS technologies are producing data faster than most underlying IT infrastructures can support and store (Mardis, 2011). NGS data require disk storage several orders of magnitude larger than standard biomedical data. Each step of NGS analysis generates large intermediate files, often requiring 5–10 times as much storage during the analysis phase than is required for the raw data itself. Moreover, scientists are reluctant to discard raw data, due to the continuous development of new algorithms that depend on extraction of further information from these data. Next-generation sequencing data storage and management, especially for small research groups, continue to be a major issue, due to rapidly evolving technologies producing larger and more complex data sets.

In the past, the requirement for databases in medical research confined them to traditional models of data storage that were adequately equipped for storing heterogeneous data sets. Indeed, the relational database structure represents a mature DBMS model and at the peak of its development in the 1990s was the preferred structure for some of the earliest biological databases such as the BLAST sequences and transcription factors database (Ghosh, 1990), and for clinical trial purposes the Southwest Oncology Group trials database (Blumenstein, 1989). Commercial models of relational databases including Microsoft Access (Bulusu *et al*, 2013), MySQL (Mosca *et al*, 2010) and hybrid object-relational databases such as postgreSQL and SPARQL have been widely employed in translational cancer research settings due to their open-source availability and ease of implementation (Table 1). However, relational DBMS have inflexible schemas, are not well designed for rapid growth and are prohibitively expensive for Big Data, thus their utility for NGS derived data is questionable.

In contrast to relational DBMS, non-relational database models such as NoSQL (Not Only SQL) offer high query performance, flexibility of database schema and the capacity for file-transfer across networks, albeit requiring extensive maintenance and computing power (Manyam *et al*, 2012). Different types of NoSQL databases, including document-based (e.g., MonoDB), column-family based (e.g., Cassandra or HBase) and graph-based (e.g., Bio4J) databases, have been implemented to integrate NGS with metadata. Despite their heightened requirements, NoSQLs have shown to fare better in performance tests for scalability and extensibility, as well as query retrieval times when compared with relational data models (Wang *et al*, 2014) and have, therefore, been employed for many Big Data projects.

To accommodate these database models across networks, recent years have seen the rise of cloud computing through the employment of remote or third party servers to store and process data on the Internet. Cloud-based solutions offer the advantage of heightened security, rapid scalability, dynamic allocation of services, and flexible costing, and can in principle ease collaboration between dispersed located research groups by using a shared environment on a ‘pay-as-you-go' basis (Zhao *et al*, 2013). The 1000 Genomes Project, which catalogues human sequence variation through deep sequencing of the genomes of over 1000 individuals worldwide, uses a 200 TB Amazon cloud-based data repository solution (Clarke *et al*, 2012). Commercial cloud storage solutions are also provided by Google and Microsoft, and have been used by many research institutes worldwide, namely the NIH and the European Bioinformatics Institute.

These novel storage solutions have increased the availability of cancer genomics data sets. For example, the International Cancer Genome Consortium (ICGC, 2010) and The Cancer Genome Atlas (TCGA, 2006) each store over two petabytes of genomic data across 34 cancer types. Their application to the clinic through the Mutational Signature Analysis (Alexandrov *et al*, 2013) and Pan-Cancer analysis (The Cancer Genome Atlas Research Network *et al*, 2013) studies have provided the essential link between large-scale genomics and translational research. These data sets can often be downloaded in a smaller manageable intermediate format. Services like Cancer Genomics Hub (Wilks *et al*, 2014), the Database of Genotypes and Phenotypes (NIH, 2007), the European Genome Archive (Lappalainen *et al*, 2015) and the European Nucleotide Archive (Leinonen *et al*, 2011), allow users to access, query and download regions of interest from raw large-scale sequencing data sets, whereas databases like the Catalogue of Somatic Mutations in Cancer (Bamford *et al*, 2004) and the cBioPortal for Cancer Genomics (Cerami *et al*, 2012) provide curated published data. Efforts to make these data publicly available, most notably a recent decision by the NIH to lift its restriction on the use of cloud computing (Stein *et al*, 2015), have enabled greater access to these valuable data resources by small research groups that do not have the sequencing facilities to generate these data.

Finally, cloud compute models have provided cost-effective solutions for small research groups looking to conduct sequencing analysis. These can be broadly separated into three groups, those that (1) run applications on the cloud and hide infrastructure implementation from the user; (2) provide infrastructure as a service; or (3) provide database and software as a service. One example of the latter is the Globus Genomics System (Madduri *et al*, 2014), which is an Amazon cloud-based analysis and data management client built on the open-source, web-based Galaxy platform (Goecks *et al*, 2010). The advantage of such a platform is its use of elastic scaling of compute clusters, multi-threaded parallelism of workflows and a secure file-transfer system. These features, coupled with its intuitive interface and the continuous reduction of cloud-computing costs, make it an attractive option for small research groups looking to perform short-term or modest-sized NGS projects. Alternative data management systems that allow users to integrate large-scale genomics data and various metadata are TranSMART (Athey *et al*, 2013), BioMart (Kasprzyk, 2011) and the Integrated Rule-Oriented Data System (iRODS, 2015). These platforms provide extensive modules for data integration, and have been employed globally for cost-effective, collaborative data storage for small-scale research settings.

## Ethics of genomic research: personalisation *vs* exposure

The increased adoption of genomic research in personalised medicine, particularly with the recent 100 000 Genome project, has stirred strong public debate. Genomic research poses new challenges for tissue banks and research ethics committees that may not necessarily be addressed by existing guidelines, such as the use of a tumour sample from one patient for multiple studies (due to assays now requiring smaller quantities of tissue) and the appropriate protocol for feedback of results to patients. Furthermore, studies have demonstrated the ‘re-identifiability' of apparently anonymised samples of single nucleotide polymorphism data from genome-wide association studies (GWAS). This has been achieved by searching Y-chromosome genotypes and matched demographic information in recreational genetic genealogy databases (Gymrek *et al*, 2013) or the extrapolation of individual patient disease states by matching a patient's genotype to the cohort's aggregate association results, both of which are commonly published in GWAS studies (Lumley and Rice, 2010). Coupled with the growing use of cloud-based storage systems that have eased accessibility of data on the Internet, these have led to ethical concerns surrounding the nature of participant consent in genomic research and the adequacy of current systems in protecting privacy and security.

In the UK, the NHS Health Research Authority applies legislative checkpoints governing the use of patient data or specimens, which have been adhered to through various methods. For example, biobanks use a ‘broad consent' format to address the complexity of genomic studies and to enable research use over a long time period (Hansson, 2009). The capture of tiered or selective consent has been attempted through coding systems to maximise use of data between research groups and to provide assurance that the appropriate consent has been given for their study (Ohno-Machado *et al*, 2012). Alternatively, the ‘honest broker' model has been used, in which an impartial third party performs the collection, de-identification and provision of patient data to researchers. Health information is stripped of identifiable items within the honest broker environment and assigned a research identifier, which then allows updating of clinical information as well as feedback of results to patients wherever necessary. Through the honest broker system, researchers are granted more independence and consistency in data sharing, however this comes at a logistical cost to the biobank and limits the speed at which data requests can be managed.

In the case of research databases, several methods have been used to conform to regulatory frameworks. One example is the development of de-identification algorithms that scan free-text reports and remove or encrypt identifiable information (Schell, 2006; Fernandes *et al*, 2013). These algorithms have been widely reviewed (Dhir *et al*, 2008). Another example is the linkage of a research database to the honest broker environment through their research identifiers, thus reducing the delays associated with updated data requests (Segagni *et al*, 2012). For multi-institutional databases, system-generated identifiers have been proposed to allow for institutions to use their own consent language and ethics procedures (Patel *et al*, 2007). Finally, customised user interfaces have been developed to allow users to view descriptive statistics of aggregate data according to level of authorisation – one such application is used by the Pennsylvania Cancer Alliance Bioinformatics Consortium, with views divided into ‘public query', ‘approved investigator query' and ‘data manager query' (Patel *et al*, 2006).

On-going discussions between healthcare providers, patients and government have indicated that a consensus has yet to be reached regarding best practices in governance of patient data (POST Report 474, 2014). It is however agreed that the use of the national healthcare data resource requires transparency and constant engagement with the public, as illustrated by the Department of Health's consultation for proposing new regulations of data use (Department of Health, 2014). Although this continues to be debated, research groups should operate on robust regulatory procedures that protect patient privacy, while not being overlaid with obstructive administrative barriers that may be prohibitive to research (Karp *et al*, 2008).

## An integrative research database solution for small-to-moderate-sized research groups

**Figure 1:**
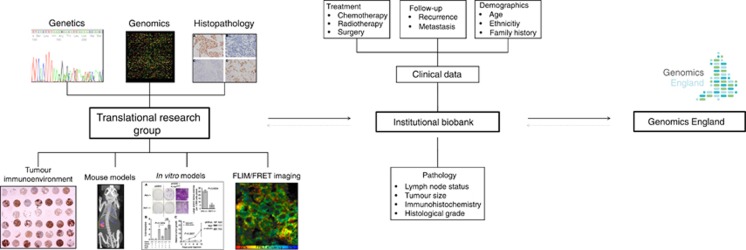
**Translational research data in the era of Genomics England.** Research data from multi-disciplinary fields such as genomics, histopathology, mouse models and fluorescence imaging as managed by a typical translational research group will be integrated with their associated clinical data managed by the institutional biobank and healthcare centre, encompassing features such as treatment, follow-up, demographic and diagnostic data. In alliance with the Genomics England project, these data and their associated biosamples will be used in GeCIP studies and fed back to both healthcare and on-going translational studies within the research group.

The Department of Research Oncology (RO) at King's College London, UK is a typical example of a moderate-sized research group working at the translational interface in breast cancer research. Over the years, a rich resource of research data has been generated from a multitude of sources, including medical records, histopathology, genomics, imaging and so on (Figure 1), as a result of its extensive involvement in experimental studies across a wide variety of platforms such as *in vitro*, *in vivo* and *in silico* (microarray and NGS) models, as well as cancer clinical trials.

Given the RO's physical location within an NHS healthcare centre, our research has also leveraged on the integration of associated patient and sample data from collaborations with the King's Health Partners Cancer Biobank (KHP-CB) and Clinical Genetics Department at Guy's Hospital. In light of a growing number of integrative projects across the department, notably the RO's forthcoming participation in the GeCIP programme, a researcher-driven database was created to facilitate the interoperability of our research. We employed a previously described CDE model (Papatheodorou *et al*, 2009) based on ISO-compliant data formats recommended by the caDSR. To conform to the data model, data normalisation was carried out on over 2000 records spanning over 200 attributes including clinical, pathological, genomic, transcriptomic, mouse model and imaging data. Normalisation involved standardisation of data formats and semantic transformation of attributes, after consultation with clinical specialists. The description of each CDE is stored in a data dictionary, which forms the ‘minimum required set' of any new data to be entered in the database. To comply with security standards, access tier was recorded to reflect the level of consent given by the patient for data use, with consent procedures set by the KHP-CB through an honest broker system. The database uses an open-source relational MySQL platform and front-end utilities for ease of querying. For larger high-throughput data from microarray and sequencing platforms, the files are stored as links in the database for querying and association with clinical data, which point to directories in our storage servers.

Taking the necessary precautions in mapping these diverse data sets to the data model and ensuring compliance with ethical standards, we believe our database presents a cost-effective, interoperable solution for the transformation of complex, heterogeneous data into actionable information for translational research from which to build a solid foundation for participating in the GeCIP endeavour.

## Harnessing Big Data in small-scale research

The move towards high-throughput translational research in cancer has led to an explosion of genomic Big Data. However, the adoption of robust yet accessible storage systems and informatics workflows in parallel with this data growth, particularly among small-to-moderate-sized research groups, has not been well documented. In this review, we have highlighted the main issues introduced by Big Data and provide a summary of potential solutions adopted by researchers to address them.

The problem of data heterogeneity has largely been dealt with by using in-house models of standardisation to fit the distinct requirements of individual research groups. These have been aided by the publication of comprehensive guides for adaptation of legacy data with modern concepts, ranging from common classes of evolution in medical vocabularies to guide data transformation (Cimino, 1998) and ways to address conceptual gaps and redundancies in data models (Richesson and Krischer, 2007). In recognition of the labour and financial costs associated with data storage, small research groups have moved toward open-source, community-driven initiatives for data management. The utility of these solutions for researchers in the Genomics England era cannot be understated, and will be instrumental for harnessing genomic Big Data in small research groups.

On-going debate about ethical use of genomic data emphasises the need for transparency in communicating genomic research to patients. Recent concerns raised by the public in response to the NHS ‘care.data' scheme exemplify the need to regain public trust on the collection and security of data for healthcare (Nature, 2014). Although previous studies have illustrated the difficulty in extracting privacy risk in genomic data, the last decade has seen renewed efforts to quantify the likelihood of an individual being identified through their genomic data using simulation tests to assess re-identifiability of data sets. This has led to the development of algorithms to prevent re-identification (Benitez and Malin, 2010) as well as stricter guidelines on the publication of GWAS data to reduce re-identification without compromising the reproducibility of the studies (Lumley and Rice, 2010). Biobanks will need to adapt to the evolution of genomic Big Data by supporting a consent infrastructure that can proactively audit donor-sharing policies and continuously track privacy risks incurred for individuals while maximising the sharing of information.

Finally, the rise of genomic data availability prompts the reminder that genomic studies and biomarkers, however advanced they may be, should be subject to the same rigorous standards and inference as any other scientific investigation. Studies of the impact of an individual characteristic or exposure, such as ethnicity, histopathological tumour type or chemotherapy on an outcome require a careful definition of the population studied, comparison groups, measurements, interventions and all other elements of a scientific clinical study, and no amount of precision or detail can correct for bias and confounding factors (Prudkin and Nuciforo, 2015). The lack of a firm strategy and well-planned study design has hindered the translation of biomarkers to clinical utility, and a growing number of publications and institutional initiatives aim to improve this issue (Staratschek-Jox and Schultze, 2010; Poste *et al*, 2015). One effective infrastructure to support a shorter way between first discovery and clinical application is a truly multi-disciplinary and multi-professional collaboration from the planning stage through to analysis and interpretation (Poste *et al*, 2015).

The decreasing cost of sequencing has improved the financial feasibility of large-scale studies such as the 100 000 Genomes Project, yet it is estimated that the cost of storing these data is not decreasing in parallel (Stein, 2010). The onus will be on individual research groups to equip themselves with the appropriate infrastructure necessary to accommodate these data. The success of these projects will in turn depend on the establishment of frameworks that incorporate accurate cancer ontology, proper study design, appropriate ethical standards and robust IT infrastructure. Overall, the challenges brought on by Big Data will enforce stronger interaction within the scientific community in using these resources effectively for translational cancer research.

## Figures and Tables

**Table 1:** Published integrative databases for cancer research

**Name of DB**	**Institution**	**Diseases**	**No of cases**	**DBMS**	**Authors**
Breast Cancer Surgical Outcomes Research Database (BRCASO)	Group Health Cooperative, Kaiser Permanente Colorado, Marshfield Clinic	Breast	6095	SQL Server	Aiello Bowles *et al.* (2012)
Pancreatic Expression Database (PED)	ICR, QMUL	Pancreas	7636	MySQL, MartView (BioMart), Perl	Chelala *et al.* (2007)
Breast Information Core (BIC)	International Agency for Research on Cancer	Breast	—	Sybase Server, SQL, PERL	Szabo *et al.* (2000)
Pathology Analytic Imaging Standards (PAIS)	Emory University	Breast, brain	4740	IB DB2 Server, SQL, XML	Wang *et al.* (2011)
Breast Diseases Registry System (BDRS)	Middle East Technical University	Breast	—	SQL Server, XML	Kocgil and Baykal. (2007)
Cooperative Prostate Cancer Tissue Resource (CPCTR)	University of Pittsburgh	Prostate	>6000	Oracle, PL/SQL	Patel *et al.* (2006)
Pennsylvania Cancer Alliance Bioinformatics Consortium (PCABC) Biorepository	University of Pittsburgh	Melanoma, breast, prostate	>11 000	NCI Cancer Biomedical Informatics Grid (caBIG), Java	Patel *et al.* (2007)
METABRIC Repository	Cambridge University	Breast	2000	CancerGrid, SQIV, SPARQL, XML	Papatheodorou *et al.* (2009)
Genes-to-Systems Breast Cancer (G2SBC) Database	Institute for Biomedical Technologies	Breast cancer	>2000	MySQL, PHP, JavaScript	Mosca *et al.* (2010)
SPORE Head and Neck Neoplasm Database	University of Pittsburgh	Head and neck	6553	Oracle, PL/SQL, Java	Amin *et al.* (2009)
GEM Registry	Cambridge University	GI	—	MS Access, SQL	Bulusu *et al.* (2013)
Cancer Gene Expression Database (CGED)	Nara Institute of Science and Technology	Breast, GI	>400	—	Kato *et al.* (2005)
OncomiR Database (OncomiRdbB)	Council of scientific and Industrial Research, India	Breast	782	MySQL, Perl	Khurana *et al.* (2014)
Stanford Translational Research Integrated Database Environment (STRIDE)	Stanford University	Various	1.3 m	Oracle, XML	Lowe *et al.* (2009)
Thoracic Oncology Program Database Project	University of Chicago	Thoracic	—	MS Access	Surati *et al.* (2011)
Georgetown Database of Cancer (G-DOC)	Georgetown University	Breast, GI	>3000	Oracle, Java	Madhavan *et al.* (2011)
Breast Cancer Gene Expression Miner (bc-GenExMiner)	Centre de Lutte Contre le Cancer Rene Gauducheau	Breast	>3000	MySQL, PHP, Java	Jezequel *et al.* (2012)
Data Warehouse for Translational Research (DW4TR)	Windber Research Institute	Breast	>5000	Oracle, AJAX	Hu *et al.* (2011)
Danish Centre for Translational Research in Breast Cancer (DCTB)	The Danish Centre for Translational Breast Cancer Research	Breast	—	—	Celis *et al.* (2003)
Cancer Genomics Hub	National Cancer Institute	Various	>11 000	XML, Apache Solr Web	Wilks *et al.* (2014)
Catalogue of Somatic Mutations in Cancer (COSMIC)	Wellcome Trust Sanger Institute	Various	—	Oracle, Biomart	Forbes *et al.* (2011)

Abbreviations: DB=database; DBMS=Database Management System; GI=gastrointestinal cancer; ICR=The Institute of Cancer Research, London; QMUL=Queen Mary University London.
